# Characteristics of Epileptiform Discharge Duration and Interdischarge Interval in Genetic Generalized Epilepsies

**DOI:** 10.3389/fneur.2018.00036

**Published:** 2018-02-19

**Authors:** Udaya Seneviratne, Ray C. Boston, Mark J. Cook, Wendyl J. D’Souza

**Affiliations:** ^1^Department of Medicine, St. Vincent’s Hospital, University of Melbourne, Melbourne, VIC, Australia; ^2^Department of Neuroscience, Monash Medical Centre, Melbourne, VIC, Australia; ^3^Department of Medicine, School of Clinical Sciences at Monash Health, Monash University, Melbourne, VIC, Australia

**Keywords:** EEG, spike-wave, duration, cluster analysis, sleep, epileptiform discharge

## Abstract

We sought to investigate (1) the characteristics of epileptiform discharge (ED) duration and interdischarge interval (IDI) and (2) the influence of vigilance state on the ED duration and IDI in genetic generalized epilepsy (GGE). In a cohort of patients diagnosed with GGE, 24-h ambulatory EEG recordings were performed prospectively. We then tabulated durations, IDI, and vigilance state in relation to all EDs captured on EEGs. We used K-means cluster analysis and finite mixture modeling to quantify and characterize the groups of ED duration and IDI. To investigate the influence of sleep, we calculated the mean, median, and SEM in each population from all subjects for sleep state and wakefulness separately, followed by the Kruskal–Wallis test to compare the groups. We analyzed 4,679 EDs and corresponding IDI from 23 abnormal 24-h ambulatory EEGs. Our analysis defined two populations of ED durations and IDI: short and long. In all populations, both ED durations and IDI were significantly longer in wakefulness. Our results highlight different characteristics of ED populations in GGE and the influence by the sleep–wake cycle.

## Introduction

The initiation of epileptiform discharges (EDs) is accompanied by intracellular paroxysmal depolarization shifts, and the termination is attributed to a process of recurrent inhibition (1). However, the factors and mechanisms responsible for the generation and termination of EDs and seizures are not well understood. Some researchers have postulated that interictal–ictal transitions depend on a Poisson process with a fixed probability of occurrence between the two states or time-dependent mechanisms characterized by a variable probability of transition based on the length of time elapsed in the current state (2).

Genetic generalized epilepsies (GGEs) are electrographically characterized by bilateral, symmetric, and generalized EDs (3). The distinction between interictal and ictal activity depends on the ED duration and associated clinical features (4). ED duration is a more objective measure, whereas whether symptoms are manifest depends on the precision of testing, particularly in relation to absence seizures (4). There is no consensus on the threshold value of ED duration to distinguish interictal from ictal activity, and 2 or 3 s is often used by researchers as the cut-off (5, 6). Although ED duration is a continuum, it is biologically plausible that there are two distinct populations of ED with relatively shorter and longer durations representing interictal and ictal activities, respectively.

Interictal EDs and high-frequency oscillations are among the important biomarkers of epilepsy (7, 8). The relationship between interictal abnormalities and seizures remains poorly understood. Studying the characteristics of seizures and EDs will help us understand the underlying rhythmicity of the complex process of epileptogenesis. Previous research has demonstrated the circadian rhythmicity of seizures and interictal ED in both focal and generalized epilepsies (9–11). The rhythmicity and patterns of seizure duration, interseizure interval, and seizure bursts in focal epilepsy have also been described (12, 13). Many researchers have previously shown the impact of the sleep–wake cycle on epileptogenicity (10, 14). A study involving two patients diagnosed with “petit mal epilepsy” reported a unimodal or weakly bimodal distribution of durations of EDs. The same study found that discharges were relatively longer during wakefulness and shorter in sleep (15). The characteristics of ED durations and interdischarge interval (IDI) in GGE have not been well studied. A study of ED duration and IDI in relation to sleep–wake cycle will reinforce the findings of previous research by showing the modulation of ED generation. The overall assessment of multiple facets in epileptogenicity (temporal distribution of seizures, the temporal distribution of ED, ED duration, and IDI) in relation to the sleep–wake cycle is likely to provide a more thorough understanding of this complex process.

Against this backdrop, we sought to investigate (1) the characteristics of ED duration and IDI and (2) the influence of vigilance state on ED duration and IDI. We hypothesized that ED duration and IDI are of bimodal distribution, and the characteristics of those populations are influenced by the sleep–wake cycle.

## Patients and Methods

### Case Ascertainment and EEG Data Acquisition

We have previously published our methodology (16, 17). In brief, we prospectively recruited patients through consecutive referrals from epilepsy clinics at two tertiary hospitals in Melbourne, Australia (St. Vincent’s Hospital and Monash Medical Centre). The International League Against Epilepsy criteria were used to establish the diagnosis of GGE (18, 19).

24-h ambulatory EEGs were recorded in all subjects according to the standard protocol as described previously (16, 17). We used the 32-channel, Compumedics Siesta ambulatory EEG system (Compumedics Ltd., Melbourne, Australia). Patients were advised to have their natural nocturnal sleep during the recording. One reader (US) reviewed all EEG recordings using ProFusion 4 software (Compumedics Ltd., Melbourne, Australia) on the longitudinal bipolar montage with 0.5–70 Hz bandwidth. A poor quality EEG recording with signal dropouts and artifacts was a criterion for exclusion. Normal EEGs without any EDs were also excluded from the analysis.

All EDs were assessed for characteristics including duration, time of onset, and the state of vigilance. We followed the American Academy of Sleep Medicine (AASM) guidelines to determine the state of vigilance (wakefulness versus sleep) and the wake–sleep boundary (20). Sleep stages were classified into NREM and REM sleep based on the AASM criteria. However, we did not subclassify NREM sleep into stages N1, N2, and N3. The sleep onset was defined as the transition from stage W (wakefulness) to any sleep stage.

For our analysis, we had to determine the state of vigilance (awake, NREM sleep, and REM sleep) when the ED emerged. We followed the AASM rules in coding. For example, when we detected a discharge in an epoch satisfying criteria for stage N1, N2, or N3, we scored it as an ED during NREM sleep. If we detected alpha rhythm anytime during the epoch with body movements, it was coded as W (wakefulness). In keeping with the AASM rules, when the preceding or ensuing epoch was scored as W, the epoch with body movements was scored as wakefulness. Otherwise, we considered the epoch in question to be in the same stage as the following epoch (20).

Epileptiform discharges were defined as generalized polyspikes, polyspike-wave, and spike-wave occurring in the forms of a single discharge or a burst. The duration of discharges was measured manually with a tool provided in the EEG software. ED duration was defined as the distance from the beginning of the first spike or polyspike to the end of the last wave and expressed in seconds. The point at which the first appreciable amplitude change of the spike or polyspike from the baseline occurred was taken as the onset. The IDI was measured from the end of an ED to the beginning of the next ED and expressed in minutes. We entered the EEG and clinical data into a custom-made electronic database. In the current analysis, we included patients who had counts of ≥100 generalized EDs captured on 24-h EEG to ensure that we had a sufficient number of events for statistical analysis.

### Statistical Analyses

We followed the methodology previously adopted by us, as summarized below, to study the seizure duration and interseizure interval in focal epilepsy (12). We sought to characterize populations of ED duration and IDIs. We used the K-means cluster analysis and finite mixture modeling (FMM) independently to quantify and characterize the subpopulations of ED duration and IDI (12). We used seconds as our metric to study ED durations. As IDIs were generally longer, we used minutes to measure the intervals. We believe that this statistical methodology is applicable to both focal and generalized EDs as we analyzed the distribution of a series of continuous variables.

On the basis of the biological plausibility and previous research, as detailed below, we hypothesized the existence of two populations of EDs. ED duration is a continuous measure divided into “interictal” and “ictal” groups based on the clinical features of seizures. In GGE, myoclonic seizures and generalized tonic–clonic seizures have distinct ictal rhythms to help this differentiation (4). The recognition of ictal EEG in absence seizures is more challenging when clinical features are not observed. Hence, researchers have used a threshold value of 2 or 3 s of ED duration to make the distinction between “interictal” and “ictal” EEG of absence seizures (5, 6). Therefore, we hypothesized that it is plausible to exist two populations: ED with relatively short and relatively long durations. Our hypothesis was supported by previous research demonstrating a bimodal distribution of ED durations (15).

As the first step, we used histograms and kernel density plots of ED durations and IDI to delineate subpopulations and ensure that our hypothesis was plausible. Then we used K-means cluster analysis and FMM to quantify those subpopulations. The cluster analysis was used as an exploratory tool independent of the distribution of data, whereas FMM assumed an underlying normal distribution with a statistical target. Good agreement between the two approaches was strong evidence supporting the existence of distinct populations. For each population in each subject, we reported mean, SD, and the relative prevalence. These values provided further evidence to characterize populations. For example, small SDs and large clusters indicated definite populations, whereas large SDs and small clusters (prevalence < 10%) increased the possibility of spurious populations (12).

In addition, we calculated mean ED durations and IDI of all subjects collectively at hourly bins on the military time scale. We then generated scatter plots to study the changes of mean durations across the 24-h time scale.

Having defined populations of ED duration and IDI, we then sought to study the influence of vigilance state (sleep versus wakefulness) on these two variables. We calculated the mean, median, and SEM in each population collectively from all subjects for sleep state and wakefulness separately. We used a similar methodology to study the influence of seizure-free duration and AED therapy. Seizure-free duration was defined as the time gap between the last seizure prior to the EEG recording and the date of EEG. Two groups were defined for comparison: seizure-free duration <2 years and ≥2 years. Four groups were defined according to the number of AEDs used (0, 1, 2, and 3). We then used Kruskal–Wallis test to compare the differences among groups.

Finally, we calculated the ED rate (number of EDs per hour) for sleep and awake states separately. We conducted the data analyses with Stata (version 13.1) statistical software package (StataCorp LP, TX, USA). *P* < 0.05 was deemed significant.

This study was approved by the Human Research Ethics Committees of St. Vincent’s Hospital and Monash Health. We obtained written informed consent from all participants included in the study.

## Results

We performed 24-h ambulatory EEGs on 120 patients diagnosed with GGE, and 107 recordings were abnormal showing EDs. We analyzed data from 23 subjects who had 100 or more generalized EDs during the 24-h period. The clinical and demographic characteristics of this cohort are summarized in Table 1. The mean sleep duration was 9.4 h (SD = 1.5), whereas the mean sleep onset and offset times were 2249 and 0735 h, respectively. No EEGs had to be excluded from the analysis due to poor recording quality.

**Table 1:** Summary of demographic and clinical characteristics of the subjects.

ID	Age (years)	Sex	Syndrome	Antiepileptic drugs	Number of ED/24 h	Seizure-free duration (days)	Duration of epilepsy (years)
1	23	F	JAE	VPA, LTG	112	1	10
2	42	F	JAE	LTG	198	730	31
3	26	F	JAE	VPA, LTG	214	60	15
4	45	M	JME	VPA, LEV	215	730	28
5	30	M	JAE	VPA, LEV	319	1	18
6	20	F	JAE	VPA, LTG	134	4	9
7	36	F	JAE	VPA	101	730	20
8	28	M	JME	VPA	286	1278	10
9	22	F	JME	LTG, LEV	271	32	9
10	28	F	CAE	VPA, ZON	163	1	21
11	28	M	CAE	VPA	209	1	22
12	30	F	JAE	–	243	180	15
13	19	M	JAE	VPA, LTG	177	180	1
14	25	F	JME	LTG	133	1	11
15	33	M	JAE	–	116	15	8
16	57	F	JAE	VPA	194	510	33
17	33	F	JAE	LEV	125	3650	22
18	28	F	JAE	VPA, LTG, LEV	300	7	15
19	37	F	JME	VPA, LTG	203	1	24
20	19	M	JAE	VPA, LTG, ZON	236	3	6
21	44	M	CAE	ZON	301	2	39
22	20	F	JAE	LTG, TPM	162	150	7
23	34	F	JAE	LTG, TPM	267	2	20

*Note patients 12 and 15 were not on any antiepileptic medications at the time of EEG. The seizure-free duration was estimated at the time of EEG recording*.

*CAE, childhood absence epilepsy; ED, epileptiform discharges; ID, identification number; JAE, juvenile absence epilepsy; JME, juvenile myoclonic epilepsy; LEV, levetiracetam; LTG, lamotrigine; TPM, topiramate; VPA, sodium valproate; ZON, zonisamide*.

No one had clinical seizures based on the event diary and the “event button” press during the recording. We also did not detect any generalized tonic–clonic seizures or myoclonic seizures based on the EEG. As the EEGs were ambulatory, recorded in patients’ own environment, we had to depend on the diary maintained by patients and families to correlate the EEG with symptoms. In this small cohort, no one reported seizure symptoms. However, the patient-reported symptoms can be unreliable particularly in relation to myoclonic and absence seizures. With ambulatory EEG, there was no opportunity to obtain corroborative evidence of seizure activity by an experienced observer such as a doctor, a nurse, or an EEG technologist. Hence, we did not make a distinction between “ictal” and “interictal” EEG activity. We opted to refer to ED in general without making that clinical distinction.

Epileptiform discharges were significantly more frequent during sleep with the mean ED rate of 15.1/h in sleep versus 4.6 in wakefulness (*P* < 0.001).

**Figure 1:**
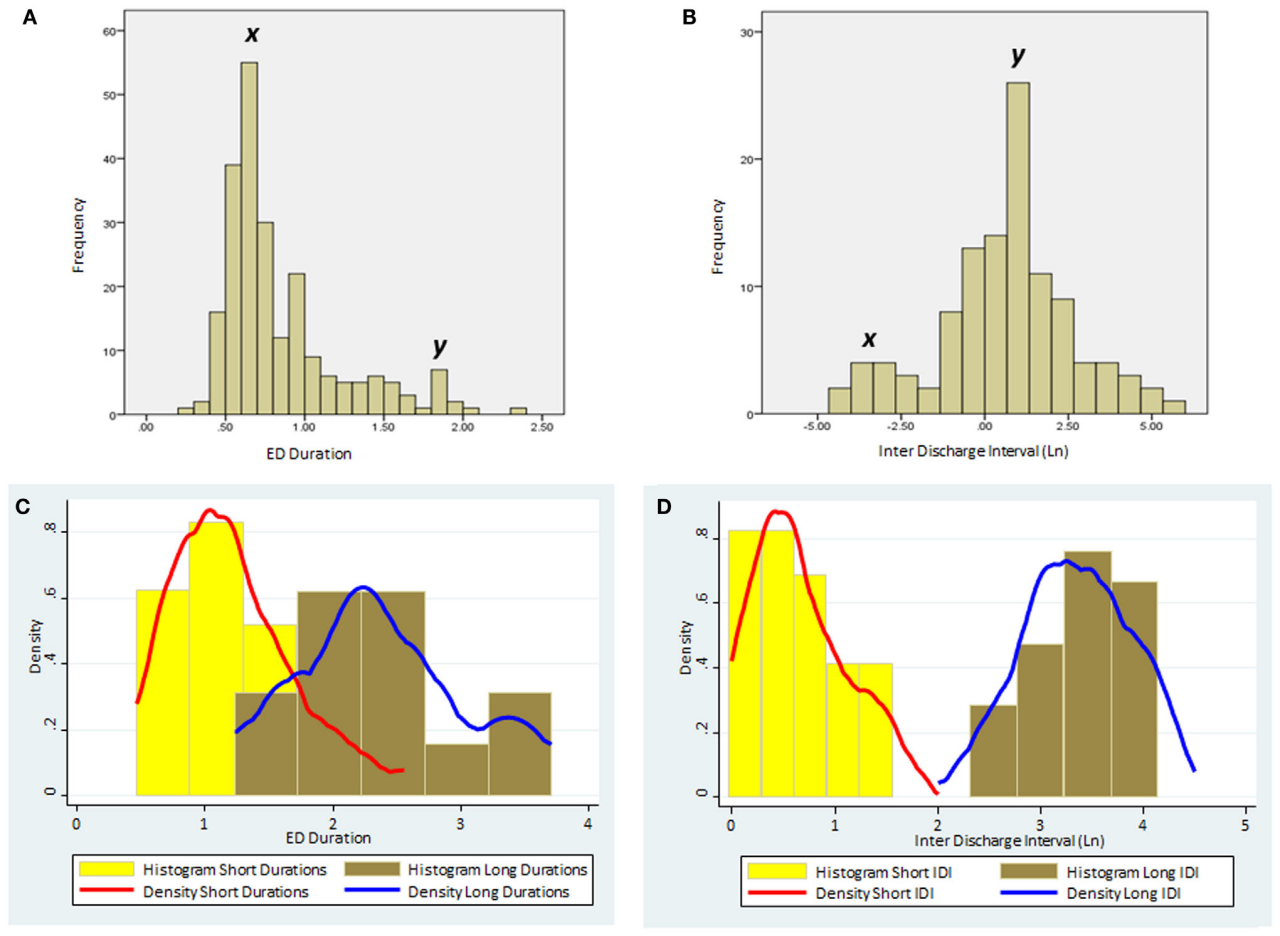
The distribution of ED duration and IDI based on the pooled data from the all 23 subjects. **(A)** Histogram of ED duration (seconds) based on the raw data. **(B)** Histogram of the IDI (minutes) based on the raw data. Note the two distinct populations in each histogram with peaks marked as *x* and *y*. **(C)** Histograms and kernel density plots of ED duration predictions jointly for the short and long duration populations detected by the finite mixture modeling. **(D)** Histograms and kernel density plots of the Ln of the IDI predictions jointly for short and long populations detected by the finite mixture modeling. (Please note that IDIs were logarithmically transformed in the histogram as the original data were skewed.) ED, epileptiform discharge; IDI, interdischarge interval; Ln, Log (natural).

K-means cluster analysis and FMM yielded concordant results. Therefore, we report only the outcome of FMM analysis here. The individual results on ED duration from the 23 subjects are summarized in Table 2. Figure 1 illustrates the histograms and kernel density plots of ED duration and IDI from the pooled data of all 23 patients. Two populations of relatively short and long durations are evident in each histogram (Figures 1A,B). The characteristics of populations revealed by FMM are illustrated in Figures 1C,D. The analysis describes two populations of ED durations: short (population 1) and long (population 2). In the majority of cases (except for subjects 3, 12, and 19), the population 1 had a higher prevalence. For example, in subject 1, the prevalence of populations 1 and 2 were 76 and 24%, respectively.

**Table 2:** Characteristics of populations of epileptiform discharge duration (in seconds).

Patient ID	Population 1 mean	Population 2 mean	Population 1 SD	Population 2 SD	Population 1 prevalence	Population 2 prevalence
1	0.858	2.485	0.220	1.636	0.76	0.24
2	0.935	1.995	0.319	0.932	0.92	0.08
3	0.913	1.826	0.240	0.645	0.44	0.56
4	1.375	2.223	0.276	0.328	0.95	0.05
5	0.659	1.235	0.218	0.403	0.66	0.34
6	1.007	4.716	0.460	2.196	0.77	0.23
7	0.695	2.397	0.307	0.544	0.79	0.21
8	1.353	2.598	0.354	0.919	0.82	0.18
9	1.355	3.074	0.341	1.230	0.93	0.07
10	2.006	4.996	0.907	2.435	0.96	0.04
11	1.779	14.027	0.704	9.952	0.99	0.01
12	0.464	1.355	0.086	0.567	0.41	0.59
13	2.553	9.042	1.311	5.158	0.66	0.34
14	1.925	11.510	0.861	10.945	0.97	0.03
15	1.159	3.709	0.326	1.490	0.73	0.27
16	1.190	13.386	0.488	8.615	0.79	0.21
17	1.235	2.397	0.361	0.952	0.81	0.19
18	1.405	4.867	0.535	2.636	0.83	0.17
19	0.539	1.701	0.113	0.925	0.36	0.64
20	3.888	14.199	2.742	8.551	0.86	0.14
21	0.849	3.332	0.389	1.829	0.90	0.10
22	0.974	7.368	0.415	3.827	0.91	0.089
23	0.989	5.498	0.318	1.860	0.73	0.27

*ID, identification number*.

Table 3 summarizes the results on IDI. Similar to ED durations, we describe two populations of IDI with short and long intervals. In all subjects, the population with short IDI had a higher prevalence.

**Table 3:** Characteristics of populations of inter-discharge interval (in minutes).

Patient ID	Population 1 mean	Population 2 mean	Population 1 SD	Population 2 SD	Population 1 prevalence	Population 2 prevalence
1	2.016	54.451	1.947	67.405	0.80	0.20
2	2.549	26.302	2.256	28.032	0.82	0.18
3	1.845	38.100	1.645	54.310	0.87	0.13
4	3.718	26.319	2.847	27.633	0.88	0.12
5	2.110	10.075	1.552	6.596	0.70	0.30
6	1.526	61.426	1.625	91.750	0.85	0.15
7	3.315	51.736	3.230	52.466	0.77	0.23
8	1.300	42.063	1.399	93.905	0.91	0.09
9	1.279	25.261	1.558	37.225	0.84	0.16
10	3.203	20.477	2.504	13.928	0.68	0.32
11	1.018	22.126	0.909	35.203	0.72	0.28
12	1.634	17.115	1.584	17.825	0.74	0.26
13	1.473	17.174	1.191	17.724	0.59	0.41
14	4.423	24.276	3.069	14.807	0.71	0.29
15	1.963	62.854	1.591	116.750	0.83	0.17
16	1.812	29.666	1.635	41.881	0.81	0.19
17	4.736	26.850	3.956	19.007	0.73	0.27
18	1.304	48.533	1.236	95.209	0.93	0.07
19	1.387	25.666	1.306	39.388	0.77	0.23
20	2.144	14.025	1.758	12.157	0.67	0.33
21	0.971	13.631	1.019	18.032	0.70	0.30
22	1.495	46.780	1.367	68.234	0.84	0.16
23	1.315	31.305	1.184	47.162	0.88	0.12

*ID, identification number*.

The influences of vigilance state on ED duration and IDI are summarized in Table 4. In this analysis, we collectively studied all discharges from all subjects dichotomized into two populations of ED durations and IDI. For each population, durations were longer in wakefulness compared with sleep state. These differences were statistically significant (Table 4).

**Table 4:** The effect of the state of vigilance on ED durations and IDIs.

State	Variable	Mean	SE (mean)	Median	Minimum	Maximum	*P* value
Awake	EDD population 1	2.560	0.621	1.740	0.647	14.970	Population 1 (sleep vs awake) = 0.001
	EDD population 2	6.806	1.169	4.515	1.264	22.425	

Sleep	EDD population 1	1.068	0.072	1.018	0.464	1.929	Population 2 (sleep vs awake) = 0.007
	EDD population 2	3.230	0.419	2.928	1.008	8.414	

Awake	IDI population 1	7.589	1.631	4.338	1.018	28.269	Population 1 (sleep vs awake) = 0.001
	IDI population 2	84.270	20.393	40.802	10.817	448.085	

Sleep	IDI population 1	2.043	0.238	1.870	0.687	4.871	Population 2 (sleep vs awake) = 0.008
	IDI population 2	30.348	4.701	20.768	7.072	78.644	

*EDD, epileptiform discharge duration (in seconds); IDI, interdischarge interval (in minutes)*.

The influences of seizure-free duration and AED therapy are detailed in Tables 5 and 6. The basic population structure of ED duration and IDI remains unchanged in the results with statistically significant differences between populations of short and long durations. This is evident at both group level and individual level. With longer seizure-free durations, ED durations tend to be shorter and IDI tends to be longer although not statistically significant. Similarly, in patients not on AED therapy, ED durations appear longer, but the sample is too skewed to draw any robust conclusions.

**Table 5:** The impact of seizure-free duration on populations of EDD and IDI.

Seizure-free duration (years)	Variable	Mean	SE (mean)	Median	Minimum	Maximum	*P* value
<2	EDD population 1	1.237	0.128	1.083	0.464	2.553	0.000
	EDD population 2	5.649	0.949	4.793	1.234	14.027	

≥2	EDD population 1	1.118	0.132	1.235	0.695	1.375	0.043
	EDD population 2	2.322	0.101	2.397	1.995	2.598	

<2	IDI population 1	1.829	0.195	1.580	0.970	4.423	0.000
	IDI population 2	31.274	3.963	25.464	10.076	62.854	

≥2	IDI population 1	3.124	0.576	3.315	1.300	4.736	0.043
	IDI population 2	34.654	5.229	26.849	26.302	51.735	

*EDD, epileptiform discharge duration (in seconds); IDI, interdischarge interval (in minutes)*.

**Table 6:** The impact of the number of antiepileptic drugs in the treatment of patients on populations of EDD and IDI.

AED number	Variable	Mean	Minimum	Maximum	Median	SE (mean)	*N*
0	EDD population 1	1.134	0.464	1.779	1.160	0.380	3
	EDD population 2	6.364	1.355	14.027	3.709	3.891	3

1	EDD population 1	1.043	0.695	1.353	1.062	0.104	6
	EDD population 2	4.351	1.995	13.386	2.498	1.816	6

2	EDD population 1	1.311	0.659	2.553	1.006	0.184	11
	EDD population 2	4.858	1.234	11.510	4.716	0.997	11

3	EDD population 1	1.260	0.858	1.518	1.405	0.204	3
	EDD population 2	4.890	2.485	7.314	4.870	1.394	3

Total	EDD population 1	1.211	0.464	2.553	1.160	0.104	23
	EDD population 2	4.926	1.234	14.027	3.332	0.794	23

0	IDI population 1	1.538	1.018	1.962	1.634	0.277	3
	IDI population 2	34.032	17.115	62.854	22.126	14.483	3

1	IDI population 1	2.447	0.971	4.736	2.180	0.574	6
	IDI population 2	31.708	13.631	51.736	28.258	5.453	6

2	IDI population 1	2.161	1.279	4.423	1.526	0.332	11
	IDI population 2	29.714	10.076	61.426	25.666	4.343	11

3	IDI population 1	1.821	1.303	2.144	2.016	0.261	3
	IDI population 2	39.001	14.025	54.451	48.527	12.605	3

Total	IDI population 1	2.110	0.971	4.736	1.812	0.222	23
	IDI population 2	32.009	10.076	62.854	26.319	3.266	23

*EDD, epileptiform discharge duration (in seconds); IDI, interdischarge interval (in minutes)*.

**Figure 2:**
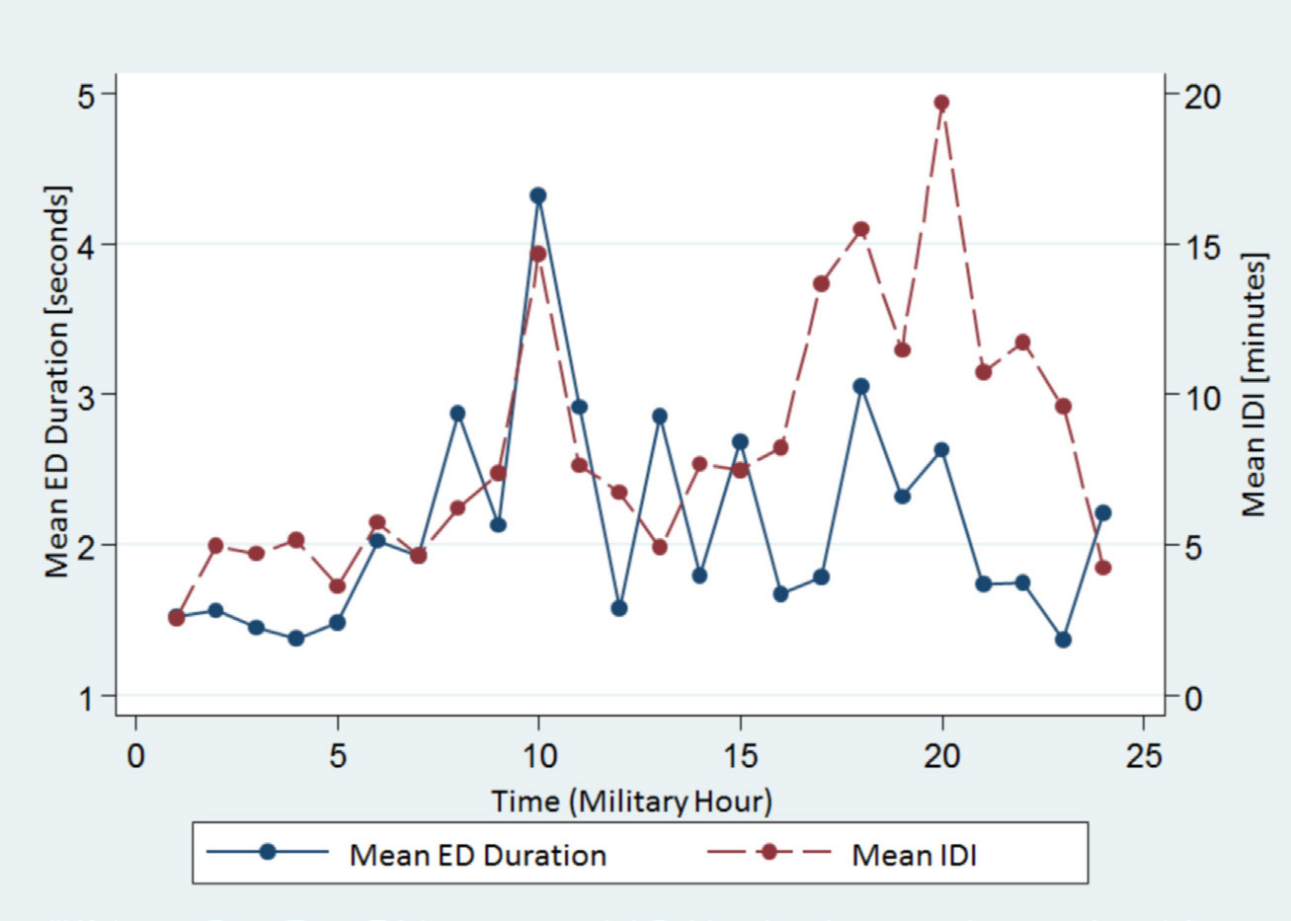
The spread of the mean ED duration and the IDI from all subjects across the 24-h time scale. Note both variables show similar undulations. ED, epileptiform discharge, IDI, interdischarge interval.

We also found that both measures varied throughout the course of the day with similar undulations. Both mean ED duration and mean IDI had two peaks at 1000 and 1800–2000 h, whereas the trough (shortest mean ED duration and ISI) was at 2300–0100 h (Figure 2).

**Figure 3:**
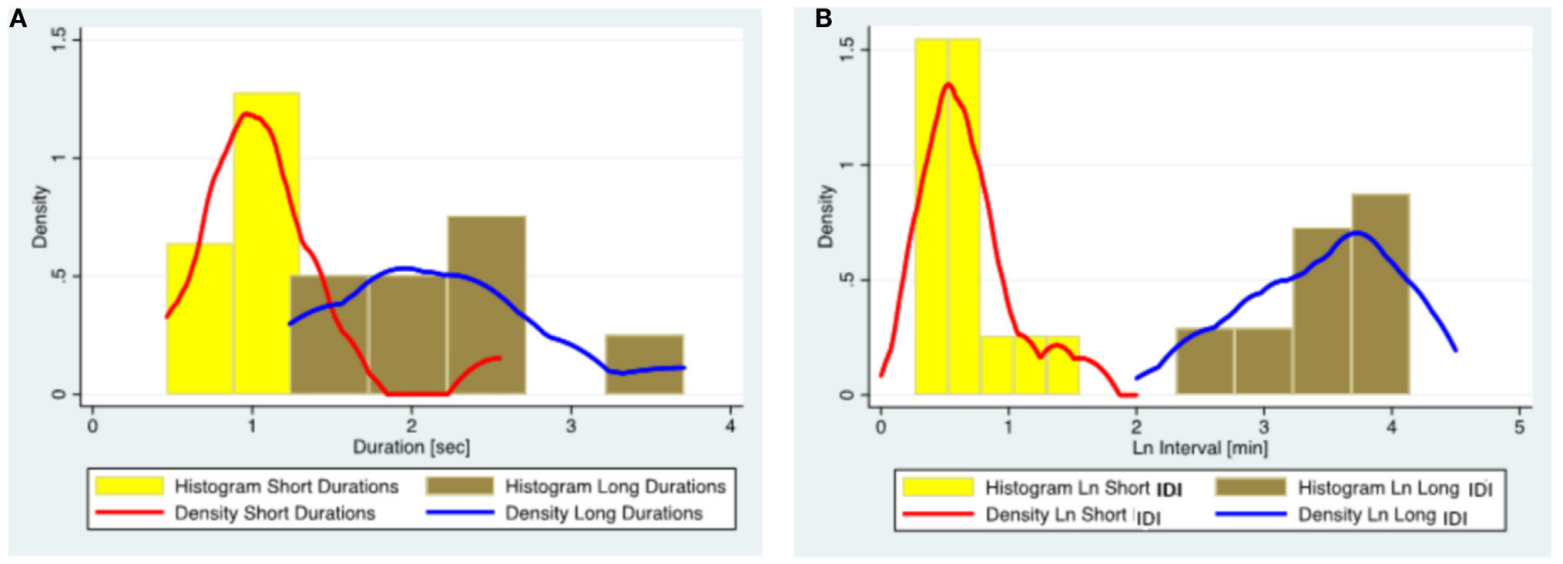
The distribution of ED duration and IDI based on the data from 15 subjects diagnosed with juvenile absence epilepsy. **(A)** Histograms and kernel density plots of ED duration predictions jointly for the short and long duration populations detected by the finite mixture modeling. **(B)** Histograms and kernel density plots of the Ln of the IDI predictions jointly for short and long populations detected by the finite mixture modeling. (Please note that IDIs were logarithmically transformed in the histogram as the original data were skewed.) ED, epileptiform discharge; IDI, interdischarge interval; Ln, Log (natural).

As our sample was heterogeneous, we conducted an additional subgroup analysis of 15 patients diagnosed with juvenile absence epilepsy (JAE). Although parameters changed, the population structure remained intact in this subgroup supporting our hypothesis (Figure 3; Table 7).

**Table 7:** Subgroup analysis of 15 subjects with juvenile absence epilepsy: the effect of the state of vigilance on EDDs and IDIs.

State	Variable	Mean	SE (mean)	Median	Minimum	Maximum	*P* value
Awake	EDD population 1	2.95	0.94	1.57	0.65	14.97	Population 1 (sleep vs awake) **=** 0.063
	EDD population 2	7.07	1.61	4.52	1.26	22.42	

Sleep	EDD population 1	1.05	0.09	0.98	0.46	1.93	Population 2 (sleep vs awake) = 0.067
	EDD population 2	3.71	0.60	3.13	1.01	8.41	

Awake	IDI population 1	7.71	2.17	3.77	1.02	28.27	Population 1 (sleep vs awake) = 0.021
	IDI population 2	75.42	15.97	62.02	10.82	188.68	

Sleep	IDI population 1	2.05	0.28	1.87	0.79	28.27	Population 2 (sleep vs awake) = 0.034
	IDI population 2	35.71	6.66	40.94	7.07	188.68	

*EDD, epileptiform discharge duration (in seconds); IDI, interdischarge interval (in minutes)*.

## Discussion

Our study shows that ED durations and IDI are clustered into populations rather than demonstrating a unimodal distribution. The populations with short ED duration and short IDI have a higher prevalence. These populations are under the influence of the state of vigilance with both the ED duration and the IDI recording significantly longer durations in wakefulness. Both variables show undulations throughout the course of the day with similar peaks and troughs. In addition, both variables demonstrate individual variations in terms of durations and prevalence of populations, potentially suggesting the individual differences in epileptogenicity. These patterns illustrate different characteristics of ED populations. Our findings strengthen the results of previous research on ED (2, 15).

Our results also highlight the influence of sleep–wake cycle on both the ED duration and the IDI. In a previous study involving patients diagnosed with GGE, we demonstrated that EDs were longer but less frequent during wakefulness while occurring in a time-of-day-dependent rhythm (11). Two previous studies on generalized epilepsy reported that a smaller number of longer EDs occurred during wakefulness, whereas the opposite trend was observed in sleep (15, 21). Stevens et al. studied 5 patients, and 2 were diagnosed with “petit mal epilepsy,” whereas Kellaway et al. studied 19 patients with generalized epilepsy (15, 21). Our current findings, using a rigorous methodology in a larger sample, are concordant with their observations, and we provide more detailed descriptions of cluster characteristics.

Kellaway et al. postulated that ED occurrence in generalized epilepsy was the result of an interaction between two processes: a 24-h circadian cycle and a shorter 100-min cycle related to sleep (21). Our findings of two populations, undulations across 24-h time scale, and changes in different states of vigilance suggest the interactions among intrinsic biorhythms.

There are potential problems linked to the identification and estimation of multipopulation data using common FMM estimation approaches. An important pitfall is the omission of a critical measure affecting the spread of data (22). We adopted a stepwise approach to overcome such pitfalls. We first used graphical aids (histograms and kernel density plots) as a guide to explore the plausibility of our hypothesis, and the plots indicated the existence of two populations with relatively short and long durations. Then, we quantified those populations independently with two approaches: K-means clustering and FMM. Both methods yielded concordant results indicating the robustness of our statistical approach.

Extracellular recordings from the somatosensory cortex of cats have revealed a bimodal distribution of interspike intervals (23). We reiterate that failure to recognize multimodal data will provide a single mean in the analysis, but the single mean will not accurately characterize the population of interest. Our analysis was directed toward an unbiased account of ED durations and IDI, with a view to identifying multimodal data. Our approach was influenced by previous research (15, 23).

Our results indicate that two populations with short and long durations of ED and IDI persist despite the influence of confounders such as AEDs and seizure-free duration. This is seen at both group level and individual level although the population parameters vary. This observation suggests that the generation of ED is modulated by the sleep–wake cycle and external factors such as AED therapy.

The distinction between “interictal” and “ictal” epileptiform activity in GGE remains contentious. In general, interictal EEG abnormalities constitute “epileptiform patterns occurring singly or in bursts lasting at most a few seconds,” whereas ictal rhythms are defined as “repetitive EEG discharges with a relatively abrupt onset and termination and characteristic pattern of evolution lasting at least several seconds” (24). When an EEG seizure pattern is not accompanied by clinical signs and symptoms, the diagnosis of a subclinical seizure is made (24). Myoclonic seizures and generalized tonic–clonic seizures demonstrate well-characterized EEG changes and clinical features; therefore, the distinction between ictal and interictal EEG activities is usually unambiguous (4). Differentiating interictal from ictal EDs can be difficult in absence seizures, as EDs demonstrate little evolution. Therefore, to differentiate ictal from interictal activity, one has to depend on the duration of the activity and accompanying clinical features, particularly impairment of consciousness, during the ED. There is no universal agreement on the duration of the GSW paroxysm constituting an absence seizure. Sadleir et al. diagnosed the absence of seizures based on the two criteria: (1) generalized spike-wave activity of any duration when accompanied by clinical signs, and (2) GSW lasting >2 seconds even if not accompanied by clinical correlates. Discharges of <2-s duration without clinical signs were identified as interictal fragments (6). Other researchers have defined a GSW burst lasting ≥3 seconds, irrespective of clinical signs, as an absence seizure (5, 25). Our study does not define the cut-off duration to distinguish between interictal and ictal activities. We were unable to correlate with clinical features as no one in the cohort reported seizure symptoms in their diaries during the EEG recording period. However, based on our finding of two populations of ED durations, one may speculate that short-duration ED populations represent interictal activity, whereas long-duration populations are ictal in nature. Further research is needed to clarify this uncertainty.

Circadian patterns of epileptogenicity have been described at the population level (14, 26). A recent study based on the long-term intracranial recordings in human focal epilepsy has found that both seizures and interictal EDs follow a circadian pattern of temporal distribution with individual differences in this rhythm (9). There is conflicting evidence on the relationship between interictal ED and seizures with studies indicating that IED may prevent seizures, facilitate seizures, or are simply an epiphenomenon (27–29). It is also unclear whether this process differs between focal and generalized epilepsies. Our study illustrates another dimension of intrinsic epileptogenicity. Understanding these multifaceted temporal patterns and dynamics of epileptogenesis at both the individual and the population levels is likely to enhance our ability of seizure prediction (30).

We acknowledge some study limitations. The sample size was small, and patients were recruited from tertiary centers introducing a bias. Our sample was heterogeneous. However, the subgroup analysis of JAE shows a similar population structure supporting our hypothesis. Most patients were on antiepileptic medications at the time of EEG recording. A drug naive, incident cohort would have been ideal for a study of this nature. Sodium valproate, levetiracetam, and lamotrigine are known to suppress EDs (31–33). Twenty of 23 patients in the cohort were treated with those 3 AEDs as monotherapy or polytherapy. Although we studied the impact of the number of AEDs used, the sample was too small and skewed to do a detailed evaluation of the influence of AED dosage. All EEGs were scored by the same reader, who was not blinded. Signal dropouts and artifacts are potential sources of error in ambulatory EEG. However, having provided written instructions to patients, we were able to obtain high-fidelity recordings.

Due to such limitations, we suggest that findings in this study should be considered preliminary. Future studies should focus on a large sample of drug naive incident cases of GGE allowing separate analysis of different syndromic subgroups with detailed sleep staging. A detailed morphologic analysis of EDs including rhythmic versus non-rhythmic and ictal versus non-ictal patterns in different states of vigilance should also be included.

In summary, distinct populations with relatively short and long durations of ED and IDI can be identified in relation to EDs in GGE. These populations are identifiable at both group and individual levels although the population parameters vary. The population characteristics are heavily influenced by the state of vigilance. These patterns highlight the characteristics of ED and IDI populations in GGE and the influence of the sleep–wake cycle on the generation of EDs.

## Ethics Statement

This study was conducted with approvals from the Human Research Ethics Committees of Monash Health and St. Vincent’s Hospital, Melbourne. All subjects provided written informed consent in keeping with the Declaration of Helsinki.

## Author Contributions

US contributed to study concept and design, literature search, data collection, data analysis, data interpretation, drafting, and critical revision of manuscript. RB contributed to data analysis and interpretation and critical revision of manuscript. MC contributed to study concept and design, data interpretation, and critical revision of manuscript. WD contributed to study concept and design, data interpretation, and critical revision of manuscript.

## Conflict of Interest Statement

The authors declare that the research was conducted in the absence of any commercial or financial relationships that could be construed as a potential conflict of interest.
